# Diagnostic performance of DNA index for detection of high hyperdiploidy in childhood B-cell acute lymphoblastic leukemia

**DOI:** 10.1371/journal.pone.0347201

**Published:** 2026-04-20

**Authors:** César Rivera-Orcoapaza, Jaime Rosales-Rimache

**Affiliations:** 1 Facultad de Salud Pública y Administración, Universidad Peruana Cayetano Heredia, Lima, Perú; 2 Servicio de Patología Clínica, Instituto Nacional de Salud del Niño San Borja, Lima, Perú; 3 Grupo de Investigación en Salud Ocupacional y Medio Ambiente, Universidad Científica del Sur, Lima, Perú; Tulane University Health Sciences Center: Tulane University School of Medicine, UNITED STATES OF AMERICA

## Abstract

**Introduction:**

High hyperdiploidy (HHD) is the most common cytogenetic abnormality in childhood B-cell acute lymphoblastic leukemia (B-ALL) and is a pivotal finding for prognosis evaluation and treatment stratification. Karyotyping is considered the gold standard for detecting HHD; however, cytogenetic cultures sometimes fail. The DNA index (DI) analysis by flow cytometry (FCM) is a quick and simple approach to estimating HHD without the need for cultures. The aim of this study was to assess the diagnostic performance of DI for detecting HHD in pediatric patients with B-ALL.

**Methods:**

A cross-sectional study was conducted on 210 pediatric patients with B-ALL at Instituto Nacional de Salud del Niño San Borja (INSNSB) in Peru between 2017 and 2021. The receiver operating characteristic (ROC) curve was analyzed using karyotype results as the reference standard to determine the optimal DI cut-off value for HHD. The main diagnostic performance indicators were subsequently described. Immunophenotypic characteristics associated with HHD were also explored using a generalized linear model (GLM).

**Results:**

A 25.2% (n = 53/210) rate of failed karyotypes was observed. DI ≥ 1.10 showed a sensitivity of 96.6% (95% CI: 93.7–99.4%), specificity of 89.8% (95% CI: 85.1–94.6%), positive predictive value of 68.3% (95% CI: 61.0–75.6%), negative predictive value of 99.1% (95% CI: 97.7–100.0%), and AUC of 0.947 (95% CI: 0.914–0.980). The overall concordance between DI and karyotype was 91.1% (n = 143/157), with a Kappa index 0.75 (95% CI: 0.62–0.87). Blast size (PR = 4.8, 95% CI: 2.7–8.5) and CD123 expression (95% CI: 1.9–73) were associated with HHD (p < 0.001 and p < 0.009, respectively).

**Conclusion:**

DI has a high diagnostic performance and is helpful as a complementary method to karyotyping for detecting HHD.

## Introduction

Detecting chromosomal abnormalities is a key laboratory finding in the prognosis and treatment stratification of childhood B-cell acute lymphoblastic leukemia (B-ALL) [1,2]. Chromosomal abnormalities can be either structural or numerical; the latter are also known as aneuploidies, which occur due to the gain or loss of one or more chromosomes from the normal diploid set. In B-ALL, High hyperdiploidy (HHD) is defined by the presence of more than 50 chromosomes, generally between 51–67, with chromosomes 4, 10, 14, 17, 18, 21, and X being the most gained [3,4]. HHD is also the most frequent chromosomal abnormality in childhood B-ALL (25–30%), is strongly associated with favorable prognoses, and is used as a classification and risk assessment criterion [5–7].

The reference method for detecting HHD is karyotyping since it allows direct visualization of chromosomes [8]. However, failed karyotypes are frequently observed in patients with hematological neoplasms, mainly due to culture failures caused by high nutritional demands, low cell proliferation rates, or sample quality-related conditions [9]. These factors produce metaphases with poor morphology, which tend to appear blurry, fused, and with ambiguous margins, making proper evaluation under the microscope impossible. Up to 50% of non-informative karyotypes have been reported in patients with B-ALL [10]. Karyotyping is also a labor-intensive technique, requiring several days for final reporting. It has low analytical sensitivity due to the limited number of metaphases analyzed (usually 20), and the quality of the result depends on the observer#39;s expertise [11,12].

DNA index (DI) is a parameter derived from the analysis of DNA content through flow cytometry (FCM) that allows the estimation of clinically significant aneuploidies such as HHD [13]. DI is obtained by comparing the DNA content of pathological and non-pathological cells present in a biological sample [14]. DNA content is measured using a nuclear-specific nucleic acid stain, like propidium iodide, which emits a fluorescent signal that can be quantified by FCM [15]. Unlike karyotyping, DI test does not require cell cultures, standing out as a faster and less labor-intensive technique, offering greater reproducibility between results and having superior analytical sensitivity, being capable of identifying aneuploid clones present in heterogeneous samples at levels below 1% [16,17].

The DI is used alongside karyotyping to identify aneuploidies with prognostic value in various hematological neoplasms; therefore, it is necessary to evaluate its validity compared to the gold standard. Our study aimed to evaluate the DI diagnostic performance in detecting HHD and explore the immunophenotypic characteristics associated with HHD to assess the contribution of FCM to the accuracy of genetic diagnosis in childhood B-ALL.

## Materials and methods

### Ethics statement

This study received ethical approval from the Institutional Research Ethics Committee of the Instituto Nacional de Salud del Niño San Borja (INSNSB) and the Universidad Peruana Cayetano Heredia (UPCH), Lima, Peru. The ethics committees determined that the study was exempt from the requirement for informed consent, as it is based on a retrospective study that utilized information from existing clinical and laboratory records, with no direct contact with patients. All patient names and other personal identifiers were removed from the database to ensure confidentiality, and only the research team had access to the database.

### Study design and participants

A retrospective cross-sectional study based on medical reports of patients diagnosed with B-ALL was conducted at the INSNSB, a specialized high-complexity hospital that provides care to pediatric patients referred from other healthcare facilities across Peru. The diagnosis of B-ALL was established according to the criteria outlined in the institutional clinical practice guidelines: Presence of 20% or more B-cell lymphoblasts in peripheral blood or bone marrow, confirmed by FCM [18]. FCM and karyotype reports processed from January 2017 (when the DI test was first implemented) to March 2021 were accessed and collected for research purposes between 25/03/2019 and 20/03/2021.

We calculated the necessary sample size to evaluate a diagnostic test performance when the true status of the condition is unknown at the time of selection. Considering that the prevalence of HHD in the pediatric population with B-ALL is approximately 25% [19,20] and anticipating test sensitivity of 90% and specificity of 80%, with a precision of 10% and a confidence level of 95%, the required sample size was determined to be 139 patients.

The final sample comprised 210 patients (S1 File). We included records of patients under 18 years old at the time of diagnosis. All patients underwent karyotype and DI analysis. We excluded cases consistent with B/myeloid and B/T mixed-phenotype acute leukemia. Immunophenotyping, DI, and karyotype studies were performed on fresh bone marrow or peripheral blood samples using K2 EDTA as an anticoagulant.

### Immunophenotype

We used FCM for immunophenotyping of at least 500,000 viable events, employing a panel of monoclonal antibodies conjugated with fluorochromes, following the EuroFlow Consortium recommendations for the diagnosis and classification of acute leukemias [21]. The precursor B-lymphoid nature of the neoplastic cells was determined based on the WHO minimum criteria [5]: strong expression of CD19 along with strong expression of at least one of the following markers: cyCD79a, cyCD22, CD10, or strong expression of CD19 along with weak expression of at least two of the following markers: cyCD79a, cyCD22, CD10. Antigen expression was considered positive when ≥20% of the cells of interest expressed the antigen in question.

The immunophenotypic characteristics selected for the study included CD66c and CD123 expression, the maturation stage (pro-B, common-B, pre-B, and mature-B) according to the European Group for the Immunological Characterization of Acute Leukemias (EGIL) classification [22], and blast size, which was calculated by dividing the geometric mean of the forward scatter (FSC) parameter of pathological B-lymphoblasts by that of total lymphocytes. Blast size was categorized for exploratory analysis as <1.35 and ≥1.35.

### DNA index

**Fig 1:**
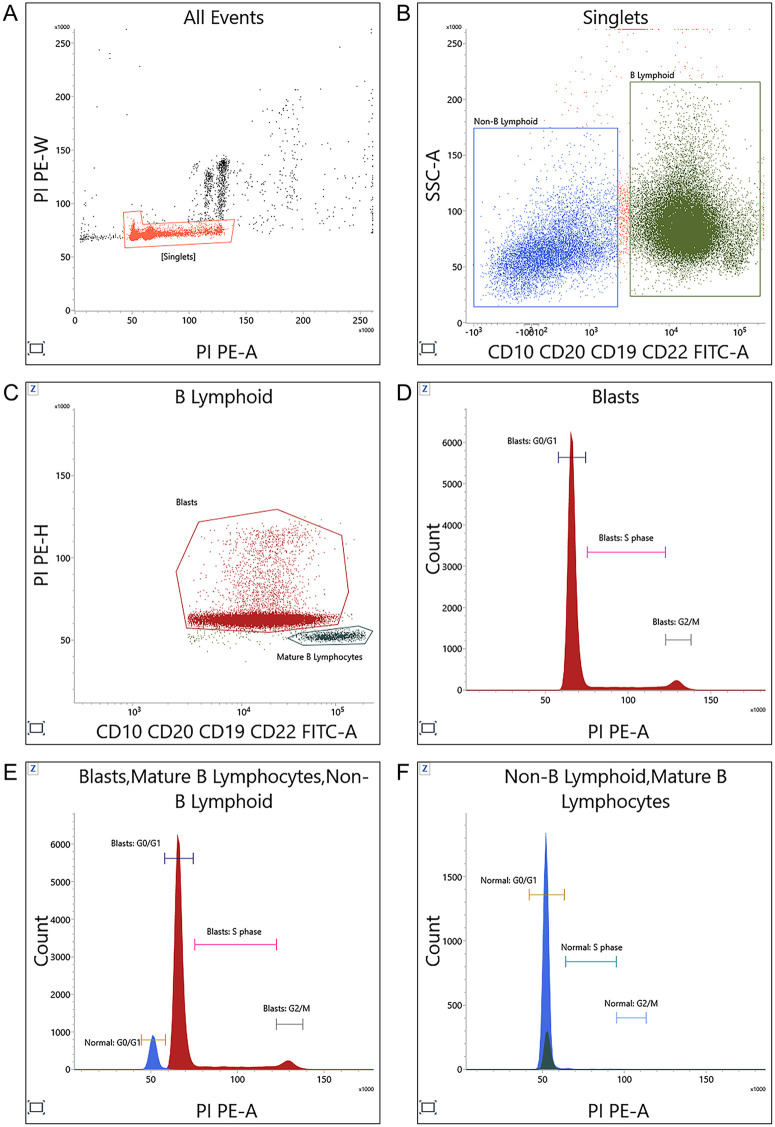
Gating strategy for acquisition, dual discrimination based on immunophenotypic, and DNA index measurement in a bone marrow sample infiltrated by B-ALL. Plot A shows the selection of viable cells from all events using the “singlets” gate, which was further applied on plot B. In plot B, all B-lymphoid cells were gated (“B lymphoid” gate) based on the positive expression of CD10, CD20, CD19, CD22, and low/intermediate side scatter. Residual non-B lymphoid cells were selected under the “non-B lymphoid” gate. Plot C displays all B-lymphoid cells and separates “blasts” from “mature B lymphocytes” based on differential fluorescence intensities of B-lineage antigens and propidium iodide (PI) staining. Histograms D and E show PI staining of the blasts and normal residual cells (non-B lymphoid and mature B lymphocytes), revealing their corresponding peaks for the G0/G1, S, and G2/M phases of the cell cycle. Overlay histogram F shows G0/G1 phase from both blasts and normal residual cells. DI was calculated by dividing the mode of the G0/G1 peak of blasts by the mode of the G0/G1 peak of normal residual cells.

**Fig 2:**
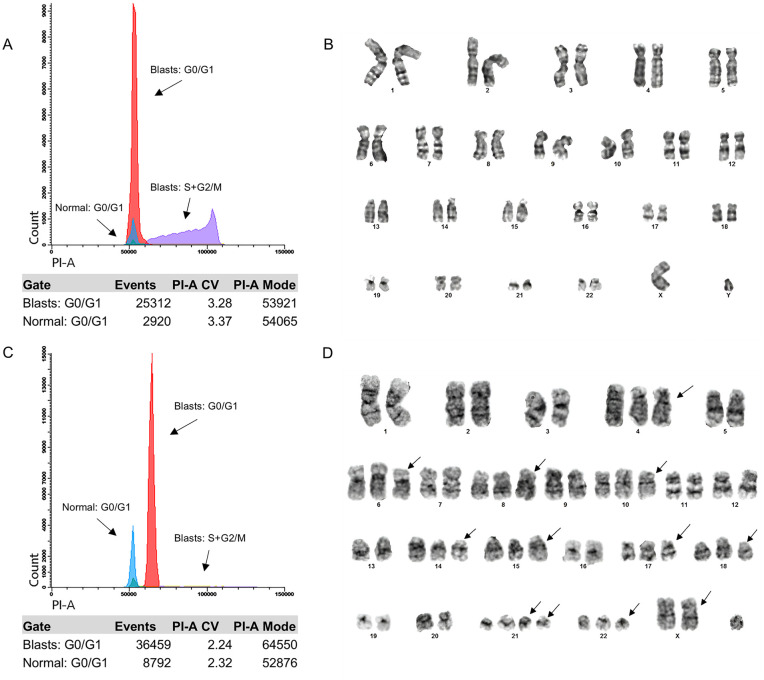
Complementarity between Flow Cytometry and Cytogenetics. A and B show DI and karyotype results of a patient with diploidy (DI: 1.00 and karyotype: 46,XY). C and D show DI and karyotype results of a patient with HHD (DI: 1.22 and karyotype: 58,XY). Arrows highlight the gain of specific chromosomes.

We used the commercial CycloscopeTM kit (Cytognos S.L, Spain), which enables the identification of B-lymphoid lineage cells within a heterogeneous biological sample. The samples were treated with a mixture of primary monoclonal antibodies against CD10, CD19, CD20, and CD22, secondary polyclonal anti-IgG antibodies labeled with fluorescein isothiocyanate (FITC), erythrocyte lysis solution, and propidium iodide for DNA staining. Cell acquisition was performed using FACS Canto II and FACS Lyric flow cytometers (BD Biosciences, USA). Multiparametric analysis was conducted using InfinicytTM software (Cytognos S.L, Spain). Both cytometers underwent daily quality control using CS&T beads (BD Biosciences, USA), with monthly voltage calibration and compensation. DI was calculated by dividing the modal fluorescence intensity for propidium iodide in pathological cells in the G0/G1 phase by the modal fluorescence intensity for propidium iodide in residual normal cells in the G0/G1 phase (Figs 1 and 2).

### Karyotype

Karyotyping was conducted from cytogenetic cultures of bone marrow and peripheral blood using Marrow Max medium, with incubation for 24–48 hours. The GTG banding technique (trypsin digestion and Giemsa staining) was employed, and the analysis was performed by qualified personnel from the Genetics department, with a second review conducted by a different specialist. The modal chromosome number, the most frequently observed chromosome count across all analyzed metaphases, was recorded. The results were categorized as hyperdiploid karyotypes (>50 chromosomes) and non-hyperdiploid karyotypes (≤50 chromosomes).

### Statistical analysis

The clinical and biological characteristics of the population were described, and differences in the frequency of HHD were evaluated using Fisher exact test. The Mann-Whitney U test was used to compare DI and blast size values between hyperdiploid and non-hyperdiploid karyotypes. ROC curve and AUC were analyzed using karyotype results as the reference standard. The optimal cut-off point was determined by selecting the DI value with the highest overall classification accuracy that maximizes both the sensitivity and specificity simultaneously. Diagnostic performance indicators were described, and the results of both tests were compared using overall concordance and kappa index. An exploratory analysis of immunophenotypic characteristics associated with HHD was performed using nested GLM (generalized linear model) with Poisson distribution family and log-link function. Additionally, DI value was evaluated in patients with other types of aneuploidies using linear regression. For all hypothesis tests, a p-value <0.05 was considered statistically significant. Data was analyzed using STATA software version 15.0 (StataCorp LLC, College Station, TX, USA).

## Results

The clinical and biological characteristics of the study population are shown in Table 1. A total of 210 pediatric patients with karyotype and DI studies were included in the analysis. Ages ranged from 3 months to 17 years. A slight predominance of males was observed (male-to-female ratio = 1.5:1). Karyotype and DI studies were performed using bone marrow (58.1%) and peripheral blood (41.9%). 25.2% of the karyotype reports were non-informative due to failures in cultures. Among the informative karyotype (157/210), HHD was confirmed in 29 cases (18.5%). A predominance of the common B phenotype was observed (75.2%), along with a high frequency of aberrant expression of CD123 (66.7%) and CD66c (61.9%). Co-expression of CD123 and CD66c was observed in 50.7% of the cases. No statistically significant differences were found in karyotype, DI, or other immunophenotypic characteristics between males and females (see S1 Table).

**Table 1:** Clinical and biological characteristics of the population (n = 210)^†^.

	n (%)
**Sex**	
Female	85 (40.5)
Male	125 (59.5)
**Age (groups)**	
< 1 year	9 (4.3)
1 to 10 years	155 (73.8)
> 10 years	46 (21.9)
**Maturation stage**	
Common-B	158 (75.2)
Pre-B	40 (19.1)
Pro-B	12 (5.7)
**CD123 expression**	
Negative	69 (33.3)
Positive	138 (66.7)
**CD66c expression**	
Negative	80 (38.1)
Positive	130 (61.9)
Blast size*	1.18 (1.06-1.35)
DNA index*	1.02 (1.00-1.14)
**Karyotype**	
≤ 50 chromosomes	128 (81.5)
> 50 chromosomes	29 (18.5)

* Median (IQR), IQR: interquartile range (p25-p75).^†^ Some variables may rise to less than 210 due to missing data.

Hyperdiploid karyotypes were associated with higher DI values (p < 0.001), which ranged from 1.09 to 1.59. Increased expression of CD123 and CD66c was observed in hyperdiploid karyotypes compared to non-hyperdiploid karyotypes (27.2% vs. 1.9%, p < 0.001, and 25.7% vs. 5.4%, p < 0.001, respectively). The presence of HHD was related to larger leukemic blast sizes. No cases of HHD were identified in patients under 1 year of age (p = 0.022) (Table 2). Hyperdiploid karyotypes were also associated with the presence of structural cytogenetic abnormalities (p = 0.007) and the absence of molecular genetic alterations (p = 0.003), independent of their prognostic significance (see S2 Table).

**Table 2:** Frequency of HHD according to clinical and biological characteristics (n = 157)^†^.

	Karyotype		
	≤50 chromosomes (n = 128)	>50 chromosomes (n = 29)	p
	n (%)	n (%)	
**Sex**			0.3
Female	48 (77.4)	14 (22.6)	
Male	80 (84.2)	15 (15.8)	
**Age (groups)**			0.022
< 1 year	8 (100.0)	0 (0.0)	
1 to 10 years	87 (76.3)	27 (23.7)	
> 10 years	33 (94.3)	2 (5.7)	
**Maturation stage**			0.331
Common-B	92 (78.6)	25 (21.4)	
Pre-B	27 (90.0)	3 (10.0)	
Pro-B	9 (90.0)	1 (10.0)	
**CD123 expression**			<0.001
Negative	52 (98.1)	1 (1.9)	
Positive	75 (72.8)	28 (27.2)	
**CD66c expression**			0.001
Negative	53 (94.6)	3 (5.4)	
Positive	75 (74.3)	26 (25.7)	
Blast size*	1.12 (1.03-1.26)	1.39 (1.28-1.47)	<0.001
DNA index*	1.01 (1.00-1.04)	1.20 (1.16-1.25)	<0.001

* Median (IQR), IQR: interquartile range (p25-p75).^†^ Some variables may rise to less than 210 due to missing data.

Recurrent molecular rearrangements were detected in 40/157 patients (S3 Table). The most frequent alterations were ETV6-RUNX1, detected in 16 patients (10.2%), followed by TCF3-PBX1 in 12 cases (7.6%), BCR-ABL1 in 7 cases (4.5%), and KMT2A rearrangements in 3 patients (1.9%). The IGH-IL3 rearrangement was identified in one patient (0.6%). No recurrent molecular rearrangements were detected in 88 patients (56.1%).

**Fig 3:**
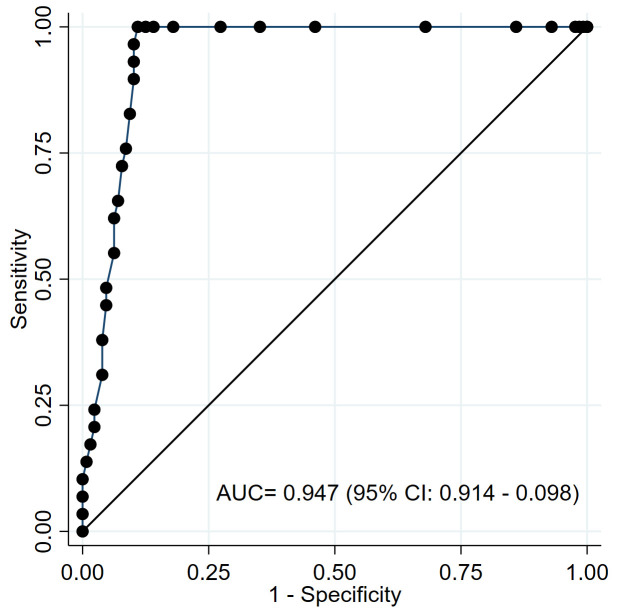
Receiver Operating Characteristic (ROC) curve of DNA index for discriminating HHD. Model showing an Area Under the Curve (AUC) of 0.947. The diagonal line represents a performance equivalent to random chance.

The analysis of the ROC curve using karyotyping as reference standard showed excellent diagnostic performance of the DI for detecting HHD (AUC = 0.947, 95% CI: 0.914–0.980) (Fig 3). The optimal cut-off value for discriminating HHD was DI ≥ 1.10, based on the highest overall classification accuracy that maximizes the others diagnostic performance metrics, achieving a sensitivity of 96.6% (95% CI: 93.7%−99.4%), a specificity of 89.8% (95% CI: 85.1%−94.6%), a positive predictive value (PPV) of 68.3% (95% CI: 61.0%−75.6%), and a negative predictive value (NPV) of 99.1% (95% CI: 97.7%−100.0%) (Tables 3 and 4). An additional age-stratified analysis confirmed consistent diagnostic accuracy of DI ≥ 1.10 across different age groups, with notably perfect sensitivity and specificity observed in patients older than 10 years (S4 Table).

**Table 3:** Comparison of DNA index and karyotype results (n = 157).

	DNA index	
Karyotype	<1.10	≥1.10
≤50 chromosomes	115	13
>50 chromosomes	1	28

**Table 4:** Diagnostic performance of DNA index ≥1.10.

Parameter	Value	(95% CI)
Sensitivity (%)	96.6	(93.7-99.4)
Specificity (%)	89.8	(85.1-94.6)
PPV (%)	68.3	(61.0-75.6)
NPV (%)	99.1	(97.7-100.0)
LR+	9.51	(5.65-15.99)
LR-	0.04	(0.01-0.26)
AUC	0.95	(0.91-0.98)
Accuracy	0.91	(0.85-0.95)
Kappa statistic	0.75	(0.62-0.87)
Youden index	0.86	–

VPP: positive predictive value, VPN: negative predictive value, LR + : positive likelihood ratio, LR-: negative likelihood ratio, AUC: area under the curve, 95% CI: 95% confidence interval.

A high overall concordance rate was observed between both tests (91.1%, n = 143/157), yiedling a Kappa coefficient of 0.75 (95% CI: 0.62–0.87, p < 0.001), while discrepancies were observed in 8.9%. A detailed characterization of the discordant cases is provided in Table 5. 13 of the 14 patients with DI ≥ 1.10 showed a diploid karyotype with 46 chromosomes and just one case presented a karyotype with 52 chromosomes but DI = 1.09. All discordant cases corresponded to the common B or pre-B immunophenotypic subtype, with uniform expression of CD123 and variable expression of CD66c. All cases with DI ≥ 1.10 and diploid karyotype, no recurrent molecular rearrangements were detected, and only one structural chromosomal abnormality was, involving del(13)(q21).

**Table 5:** Discordant cases between DNA index and conventional karyotype.

Case	Sex	Age	Maturation stage	CD123	CD66c	Blast size	Chromosomes	DNA index	Karyotype	Molecular rearrangements
1	male	5	common-B	positive	positive	–	46	1.16	46,XY	none
2	female	7	pre-B	positive	positive	1.43	46	1.33	46,XX	none
3	female	3	common-B	positive	positive	1.35	46	1.18	46,XX	none
4	male	2	common-B	positive	negative	1.37	46	1.25	46,XY	none
5	female	7	common-B	positive	positive	1.64	46	1.29	46,XX	none
6	male	4	common-B	positive	negative	1.28	46	1.25	46,XY	none
7	male	5	common-B	positive	positive	1.69	46	1.35	46,XY	none
8	male	3	common-B	positive	positive	1.34	46	1.17	46,XY	none
9	male	5	common-B	positive	positive	1.55	46	1.15	46,XY,del(13)(q21) [2]/46,XY [14]	none
10	female	5	common-B	positive	positive	1.1	46	1.22	46,XX	none
11	male	3	common-B	positive	negative	1.25	46	1.14	46,XY	none
12	female	2	common-B	positive	positive	1.11	46	1.2	46,XX	none
13	male	3	common-B	positive	positive	1.48	46	1.2	46,XY	none
14	female	1	common-B	positive	positive	1.12	52	1.09	52,XX, + 4, + 6, + 14, + 17, + 18, + 21 [6] / 46,XX [24]	none

It was found that an FSC blast/lymphocyte ratio ≥1.35 is associated with a 5-fold higher likelihood (95% CI: 3.0 to 8.3) of having a karyotype with >50 chromosomes or DI ≥ 1.10 compared to FSC ratios <1.35 (p < 0.001). Similarly, the aberrant CD123 expression was associated with a 15-fold higher likelihood (95% CI: 2.3 to 96.6) of having HHD based on karyotype or DI (p = 0.004). On bivariate analysis, CD66c expression was associated with HHD (PR = 3.8, p < 0.001); however, this association was not statistically significant in multivariate analysis (PR = 1.06, p = 0.845). No association with the maturation stage was observed in multivariate model (Table 6).

**Table 6:** Immunophenotypic characteristics associated with HHD based on karyotype or DNA index.

	Crude model			Adjusted model*		
	PR	95% CI	p	PR	95% CI	p
**Maturation stage**						
Common-B	Ref.			Ref.		
Pre-B	0.39	0.17 - 0.91	0.029	0.76	0.40 - 1.43	0.391
Pro-B	0.26	0.39 - 1.72	0.161	0.22	0.04 - 1.29	0.094
**Expression of CD123**						
Negative	Ref.			Ref.		
Positive	28.0	3.9 - 199.0	0.001	15.0	2.3 - 96.6	0.004
**Expression of CD66c**						
Negative	Ref.			Ref.		
Positive	3.77	1.88 - 7.55	<0.001	1.06	0.61 - 1.84	0.845
**Blast size**						
<1.35	Ref.			Ref.		
>=1.35	7.36	4.51 - 11.98	<0.001	4.98	3.01 - 8.25	<0.001

*Adjusted for maturation stage, CD123 expression, CD66c expression, and blast size. PR: prevalence ratio. 95% CI: 95% confidence interval.

**Fig 4:**
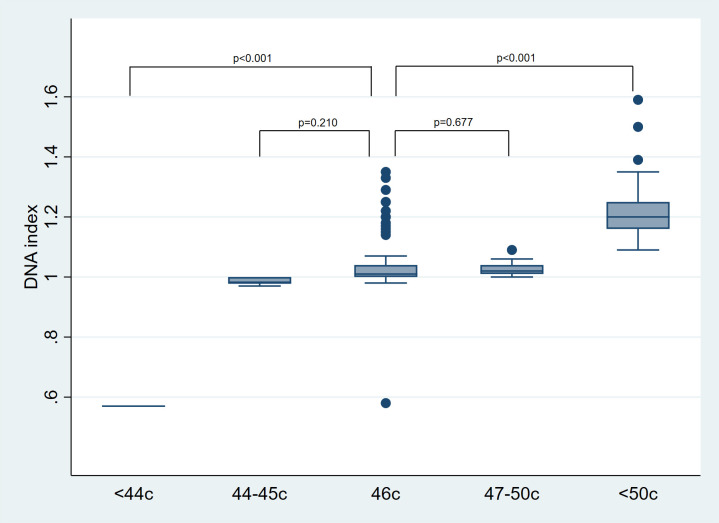
Comparison of DNA index by aneuploidy status. Low Hypodiploidy (<44c), High Hypodiploidy (44-45c), Diploidy (46c), Low Hyperdiploidy (47-50c), High Hyperdiploidy (>50c). DI values from patients with minor chromosomal gains or losses (High hypodiploidy and Low hyperdiploidy) showed no significant differences compared to diploid patients. A high degree of variability was observed in the DI values within the Diploid group.

The linear regression analysis showed that DI is primarily associated with aneuploidies of prognostic significance in B-ALL (Table 7 and Fig 4). Low hypodiploidy (<44 chromosomes) was associated with lower DI values, being 0.47 units lower compared to diploidy (reference category). In contrast, high hypodiploidy (44–45 chromosomes) and low hyperdiploidy (47–50 chromosomes) did not show significant differences compared to diploidy (MD = −0.05, p = 0.210 and MD = −0.01, p = 0.677, respectively). Conversely, high hyperdiploidy (>50 chromosomes) showed a significant increase in DI values, with an average of 0.19 units higher compared to diploidy (95% CI: 0.16–0.23, p < 0.001).

**Table 7:** Association between DNA index and other aneuploidies.

	n	mean	sd	MD	95% CI	p
**Aneuploidies**						
Diploidy (46c)	100	1.04	0.09	Ref.		
Low Hypodiploidy (<44c)	1	0.57	0	−0.47	−0.64 to −0.29	<0.001
High Hypodiploidy (44-45c)	5	0.99	0.01	−0.05	−0.13 to 0.03	0.21
Low Hyperdiploidy (47-50c)	22	1.03	0.02	−0.01	−0.05 to 0.03	0.677
High Hyperdiploidy (>50c)	29	1.23	0.11	0.19	0.16 to 0.23	<0.001

sd: standard deviation. MD: mean difference. 95% CI: 95% confidence interval.

## Discussion

This study aimed to assess the diagnostic performance of the DI and determine the most appropriate cutoff value for detecting HHD, defined as the presence of >50 chromosomes in the karyotype. Our findings demonstrated that a DI ≥ 1.10 is effective for identifying HHD in pediatric patients with B-ALL, showing a sensitivity of 96.6%, specificity of 89.8%, PPV of 68.3%, and NPV of 99.1%. These results are consistent with previous studies that have described a correspondence between DI values of 1.10 and karyotypes with more than 50 chromosomes [23–25].

For the selection of the optimal DI cutoff point, both statistical performance and clinical applicability were considered. The decision was initially based on the principle of maximizing sensitivity and specificity simultaneously. However, given that an increase in one parameter almost always results in a decrease in the other, it was necessary to prioritize sensitivity while allowing for an acceptable reduction in specificity. In clinical practice, sensitivity would be more important than specificity due to the high prevalence of HHD in pediatric patients with B-ALL. Furthermore, as the DI test is a faster and relatively less expensive technique compared to karyotyping, its utility aligns more closely with screening tests, where sensitivity is prioritized.

Based on the data obtained from the ROC curve and diagnostic validity parameters, selected cutoff point demonstrated an optimal balance between sensitivity and specificity, with an overall classification accuracy of 91.1%, surpassing all other cut-offs. Additionally, the positive likelihood ratio (LR+ = 9.51) indicates that a DI result ≥1.10 significantly increases the probability that patients have more than 50 chromosomes in their karyotype. Conversely, the negative likelihood ratio (LR- = 0.04) was very low, suggesting that a value below the cut-off substantially reduces the probability of having the condition of interest.

DI exhibited high sensitivity, successfully detecting HHD in nearly all cases confirmed by karyotyping (n = 28/29). Cut-off point ≥1.10 was only insensitive in one case (karyotype 52,XX with DI = 1.09). This discrepancy may be attributed to methodological constraints in detecting the gain of small chromosomes or genetic material that is insufficient to cause significant changes in total DNA content [20,26].

The specificity of DI was relatively lower due to a higher number of discrepancies in the group of patients with non-hyperdiploid karyotypes (n = 13/128). Notably, 11 of these 13 cases exhibited DI values ≥1.16, which suggests that several discordant cases may reflect limitations of conventional karyotyping, including potential false-negative results. As orthogonal confirmatory techniques such as fluorescence in situ hybridization (FISH) or chromosomal microarray analysis were not available in this study, additional genomic profiling to confirm or exclude true HHD of these discrepant cases could not be performed. We recommend interpreting the specificity observed here with caution due to the limitation of relying solely on karyotyping as the reference standard. We suspect the presence of false negatives in karyotyping, likely due to its low sensitivity in detecting minor aneuploid clones and clonal selection during cultures, where non-aneuploid minor subclones may be more prone to cytogenetic growth than the predominant aneuploid clone [20,27].

A strong correlation was observed between karyotype and DI results, with an overall concordance rate of 91.1%. These findings are consistent with most published studies [23–25,28,29], highlighting the utility of DI as a complementary tool to karyotyping for diagnosing aneuploidies. The Kappa coefficient was 0.75, suggesting substantial agreement between both methods beyond chance. Using a dual discrimination approach based on lineage-specific B-lymphoid monoclonal antibodies likely enhanced DI accuracy by minimizing interference from other residual cell populations typically present in peripheral blood and bone marrow samples [30].

The percentage of discrepant results between karyotype and DI was 8.9%, which is consistent with other studies [24,28,31]. These discrepancies occur because both techniques rely on different principles to estimate chromosomal gains and losses. DI analysis is based on measuring a fluorescent signal that correlates with the total amount of DNA in the cells, rather than direct chromosomal observation as in karyotyping. In this context, certain cytoplasmic components, such as mitochondria (which contain additional genetic material), the loss of small chromosomal fragments (such as deletions), gene amplification due to drug resistance, or polyclonal leukemias with varying DNA content, can contribute to discrepancies in aneuploidy estimates when using DI [32,33].

In line with the above, it is also essential to improve the standardization of procedures in FCM laboratories to prevent significant variability in DNA content measurement, particularly when working with values close to the diagnostic cutoff. To achieve this, laboratories should establish consensus-based technical guidelines that include recommendations on critical aspects during analysis of DNA content. For example, selected fluorochrome must bind specifically to nucleic acids, exhibit strong fluorescence, and display a stoichiometric relationship with DNA content [34]. Accordingly, DNA-selective fluorochromes are favored, whereas minimal RNA binding can be mitigated with RNase treatment. Furthermore, the implementation of internal controls such as fluorescence standards, diploid reference cells, exclusion of cell aggregates and debris, and verification of the coefficient of variation (CV) of G0/G1 peaks are essential for ensuring the reliability of results [35,36]. Cross-validation between laboratories should also be encouraged through interlaboratory comparison exercises and active participation in external quality assessment programs.

Our findings also demonstrated that the DI test is particularly valuable in failed or non-informative karyotypes. While karyotyping relies on the ability of cells to divide and produce metaphase chromosomes suitable for microscopic analysis, DI does not require cell cultures, as it can assess DNA content at any phase of the cell cycle. This makes it a rapid, cost-effective, and culture-independent approach for estimating the presence of clinically significant aneuploidies such as HHD.

In our study, the karyotype failure rate was 25.2%. Typically, the failure rate for karyotyping in hematologic neoplasms ranges from 10% to 20% [37,38], although case reports can vary widely depending on laboratory conditions; some studies have reported failure rates exceeding 50% [10,39]. We believe that the high rate of failed karyotypes observed in our study is more closely related to the quality of the samples submitted to the laboratory than to technical errors during processing. Several authors have emphasized the importance of maintaining appropriate pre-analytical conditions when performing cell culture studies. For instance, Javed et al. found that 31.2% of culture failures are due to poor sample quality, followed by 20% related to ongoing treatment [40]. Additional contributing factors include, but are not limited to, prolonged sample preservation time, too low or high cell counts, poor mitotic index in bone marrow specimens, sample volume, diagnostic type, and other conditions related to sample processing [41,42].

Despite the high occurrence of failed karyotypes in our study, this does not appear to have compromised the validity of the results. A subsequent analysis of our dataset demonstrated that culture failure was not associated with DI, thereby minimizing the likelihood of bias due to selective information loss (S5 Table).

An effective approach to overcoming the issue of failed cultures is the combined application of DI and another test that can be used on interphase cells like FISH with centromeric probes, or multiplex ligation-dependent probe amplification (MLPA). For example, Reyes-Núñez et al. simultaneously applied DI and MLPA—a rapid and highly specific technique to detect gains or losses of genetic or chromosomal regions—and found a significantly higher proportion of aneuploidies compared to the detection rate obtained by each method individually (up to 71.7%) [43]. In turn, Yu CH et al. reported that both DI and MLPA strongly correlate with aneuploidy status, demonstrating greater sensitivity than conventional cytogenetic analysis [44]. Other studies have also described a good correlation between karyotyping and MLPA (R² = 0.7829), with the latter even capable of detecting a greater number of chromosomes [45].

Other advanced molecular techniques that can be employed include comparative genomic hybridization (CGH), single nucleotide polymorphism (SNP) arrays, and optical genome mapping (OGM) [46]. These methods have been shown to enhance the detection of aneuploidies, providing specific information about the chromosomes involved and additional structural abnormalities; however, high cost and complexity limit their cost-effectiveness, particularly in resource-constrained settings.

The underlying genetic and chromosomal abnormalities in many types of leukemia are often reflected in the immunophenotype. For instance, an increase in DNA content is typically associated with a corresponding increase in blast size. The presence of large, pleomorphic blasts has been described as indicative of hyperdiploidy and polyploidy in acute myeloid leukemia [47]. In our study, we found that increased blast size (FSC blast/lymphocyte ratio ≥1.35) is independently associated with a higher likelihood of hyperdiploid karyotypes and/or DNA content. Some studies even recommend adjusting DI analysis by the FSC parameter to improve the diagnostic accuracy of aneuploidy detection through FCM [17,48].

Another immunophenotypic characteristic associated with HHD was the aberrant expression of the CD123 antigen. Overexpression of CD123 occurs due to the gain of at least one additional X chromosome in most cases. Given that the X chromosome carries the gene responsible for encoding the CD123 antigen (IL3RA gene), the genetic expression of this protein is subsequently elevated [49,50]. Furthermore, CD66c expression has been specifically associated with certain genetic abnormalities, such as BCR-ABL1, CRLF2, and HHD [51,52]. Our study initially observed a statistical correlation between CD66c and HHD; however, multivariate regression analysis showed that CD66c was not independently associated. Our findings suggest that the relationship between CD66c and HHD may be due to co-expression with other myeloid antigens, such as CD123, or simply due to its broad expression range in pediatric B-ALL.

Hypodiploidy with fewer than 44 chromosomes occurs much less frequently than HHD (approximately 1–5%); however, it is particularly notable among clinically significant aneuploidies in B-ALL because it is strongly associated with very poor prognosis. This group encompasses three aneuploid subcategories: high-hypodiploidy (40–43 chromosomes), low-hypodiploidy (31–39 chromosomes), and near-haploidy (23–30 chromosomes), with the latter two having the worst prognosis [5,53].

Despite advances in current treatment protocols, near-haploidy and low-hypodiploidy continue to exhibit a very unfavorable prognosis, with event-free survival (EFS) rates below 50% and overall survival (OS) rates below 40%, which decline even further (to as low as 9%) in relapsed childhood B-ALL cases treated under certain protocols [53–55]. A large and recent multicenter study in children with ALL that evaluated determinants of survival after first relapse identified hypodiploidy as the cytogenetic subgroup with the poorest post-relapse overall survival (14.2%), exceeding the adverse impact observed for KMT2A rearrangements and TCF3::PBX1 fusions, which showed post-relapse overall survival rates of 31.9% and 36.8%, respectively [56].

In our study, the small sample size and low prevalence were the primary limitations in establishing a valid DI cut-off point for hypodiploidy with fewer than 44 chromosomes. With a DI result of 0.57, our data identified one confirmed case of near-haploidy (karyotype with 26 chromosomes). A second patient showed a similar result (DI = 0.58), though the karyotype reported 46 chromosomes. This latter scenario could represent a false negative, likely resulting from the poor viability of the aneuploid clone or clonal selection of the non-aneuploid population during cytogenetic culture.

Near-triploidy (68–80 chromosomes) is another rare aneuploid category associated with poor prognosis. In our study, three patients classified as HHD fell into this category, showing DI values ranging from 1.39 to 1.59. It is now understood that near-triploidy arises from hypodiploid leukemic clones that undergo endoreduplication, mimicking hyperdiploidy at diagnosis [27]. Research shows that approximately 60–65% of B-ALL cases with near-haploidy and low-hypodiploidy contain duplicated clones [19]. Chromosomal duplication is characterized by a mosaic of hypo- and hyperdiploid clones coexisting simultaneously; however, the duplicated clone may be the only one detected by conventional cytogenetic methods, including karyotyping and routine FISH assays, a phenomenon known as “masked hypodiploidy.” Distinguishing true hyperdiploidy from hypodiploidy with endoreduplication poses a significant challenge in clinical management, as some patients may be misclassified, leading to potentially inappropriate risk stratification [55,57].

In this context, the DI analysis emerges as a particularly valuable tool for the identification of masked hypodiploidy in B-ALL. Recent studies have shown that, unlike true hyperdiploid or pure hypodiploid cases, hypodiploid cases with endoreduplication display a characteristic bimodal pattern on FCM–based ploidy analysis, consisting of an initial hypodiploid peak (DI =< 0.95) and a second peak corresponding to the duplicated clone, with a DI approximately twice that of the first peak. This distinctive pattern allows reliable discrimination between masked hypodiploidy and true hyperdiploidy [58].

Although molecular techniques such as microsatellite-based loss of heterozygosity (LOH) analysis and CGH/SNP microarrays provide high specificity for confirming masked hypodiploidy, their high cost, technical complexity, and limited availability restrict their routine clinical use, particularly in resource-limited settings. In contrast, FCM–based ploidy analysis represents a rapid, sensitive, cost-effective, and widely accessible approach that, when integrated with conventional karyotyping, substantially improves the detection of occult hypodiploid clones. Consistently, Gupta et al. demonstrated that combining FCM–derived DI with karyotype and targeted FISH assays for selected monosomies (chromosomes 7, 15 and 17) enables effective identification of masked hypodiploidy, even in patients with normal karyotypes or near-triploid patterns, reinforcing the role of DI as a key component of practical and scalable diagnostic algorithms [59].

Other aneuploid categories characterized by the gain or loss of a few chromosomes, such as low-hyperdiploidy (47–50 chromosomes) and near-diploidy, also called high-hypodiploidy (44–45 chromosomes), typically lack clinical significance in B-ALL. In our study, the DI test was ineffective for distinguishing these categories. We observed high variability in the DI results of the diploid group, leading to an overlap with both the high-hypodiploidy and low-hyperdiploidy groups. This resembles the findings of Bommannan et al. that identified high variability in the karyotypes of patients classified as diploid based on FCM (DI 0.95 to 1.05), with many falling within the low-hyperdiploidy range [48]. Similarly, Gupta et al. reported that the maximum discordance between DI and karyotyping occurred in the low-hyperdiploidy group (n = 10/22) and therefore, there are still issues in establishing a valid DI cut-off for accurately classifying this category [28].

Although our findings suggest that a DI ≥ 1.10 is useful for diagnosis, we did not directly evaluate its prognostic utility; therefore, appropriate validation in prospective studies is necessary. Longitudinal cohorts would allow to assess whether this cut-off can predict relevant outcomes such as early treatment response, relapse rate, and event-free survival. Moreover, such studies could establish whether the DI not only facilitates rapid and accessible detection of aneuploidy but also contributes meaningfully to therapeutic decision-making, particularly in settings where conventional cytogenetic testing is not routinely available. It would be valuable to explore the potential of combining DI with other clinical and biological markers, such as day 15 treatment response or minimal residual disease detection to improve the accuracy of current risk classification models [60–63]. Incorporating the DI into risk stratification algorithms used by cooperative groups in Latin America also represents a relevant opportunity for regional validation and standardization.

Recent evidence has strengthened the continued prognostic relevance of the DI in childhood hyperdiploid B-ALL under contemporary treatment protocols. In particular, Lee et al. conducted a large-scale analysis comparing multiple definitions of hyperdiploidy, including DI, chromosome number (51–67), trisomy of chromosomes 17 and 18, double trisomy of chromosomes 4 and 10, and triple trisomy of chromosomes 4, 10, and 17. Their study demonstrated that a DI range of 1.16–1.60 was the most favorable criterion, showing the strongest independent association with event-free survival and cumulative incidence of relapse, while identifying a substantial subgroup of patients with excellent prognosis and clear potential for treatment deintensification [64].

Some previous studies have suggested that a DI ≥ 1.10 may help differentiate risk groups [65,66], however most of the available evidence supports DI ≥ 1.16 as a more robust independent prognostic factor for event-free and overall survival, and this threshold has been incorporated into is several clinical management protocols [67–70]. In this context, our results using DI cutoff of 1.10 may represent not only an initial screening approach for HHD in settings with limited access to comprehensive cytogenetic analyses, but also as a starting point for future research aimed at evaluating clinically meaningful outcomes in the Latin American population. Furthermore, considering that FCM reports are often available before cytogenetic analysis, our threshold could serve as an early predictor of HHD for cytogeneticists, or at least as a prompt for more in-depth karyotype examination, especially when CD123 expression and/or an increased FSC blast/lymphocyte ratio are observed. A similar approach could be extended for hypodiploidy screening.

Among the limitations of our study is the potential overestimation of sensitivity or underestimation of specificity due to using an imperfect reference standard. Relying solely on karyotyping as the gold standard may be insufficient to avoid false-negative results. Several studies have reported the limited sensitivity of karyotyping in detecting leukemic aneuploid populations that are either too small or too labile for cytogenetic growth, which can be reliably identified through simultaneous DI analysis and other molecular techniques, including targeted FISH, MLPA, CGH, or chromosomal microarrays. [45,71,72].

We used karyotype reports as the sole confirmatory test due to the absence of other molecular tests for the study of aneuploidies and the need to verify the diagnostic validity of DI for routine use. For future studies aiming to evaluate performance, we recommend using a reference standard composed of at least two cytogenetic-molecular tests to confirm the absence of aneuploidies, thereby mitigating misclassification issues that can affect the accuracy of diagnostic performance estimates [73–75].

The reduction in sample size due to the exclusion of cases with failed karyotypes could also affect the accuracy of our point estimates; however, further analysis of our data revealed no significant differences in DI values between patients with informative and non-informative cytogenetics (S5 Table). On the other hand, the occurrence of failed karyotypes fully justifies the use of DI as a complementary method to karyotypes in the search for other clinically relevant aneuploidies.

Another limitation of our study is that it was conducted at a single center in Peru, which may limit the generalizability of our findings to other populations with different genetic backgrounds. We sampled patients from a pediatric referral center that receives patients from various regions across the country, mitigating representativeness within the local context; however, external validation in multicenter cohorts is warranted; in particular, studies involving Latin American populations are needed to support the applicability of the DI value across diverse ethnic and geographic contexts.

Regarding the frequency of HHD in pediatric B-ALL, it is known that prevalence rates can vary depending on the study population. For instance, most studies conducted in Western populations report an HHD prevalence of up to 35% [76,77], whereas this rate decreases to 15–25% in populations of Asian, African, and Native American descent [78,79]. In our study, 29 out of 157 patients were classified as HHD based on karyotype reports, resulting in a prevalence of 18.5%. However, an additional 11 patients with non-hyperdiploid karyotypes and 13 patients with failed karyotyping exhibited a DI ≥ 1.16. According to the current WHO recommendation, which considers both karyotype and/or DI as diagnostic criteria [5], the actual frequency of HHD in our study would be higher, at 25.2%.

The main strength of this study lies in its ability to evaluate the diagnostic performance of the DI test in comparison to karyotyping, highlighting the ability to identify cases of HHD that might otherwise have gone undetected due to failure in cultures. The results outlined here underscore the necessity of employing both methods in a complementary manner to improve the accuracy of diagnosis and prognosis in pediatric B-ALL.

## Conclusions

The DI analysis demonstrated excellent diagnostic performance in identifying HHD in pediatric patients with B-ALL. This analysis is a valuable complementary diagnostic tool to karyotyping for detecting aneuploidies, particularly in unsuccessful cytogenetic assessments. Integrating karyotyping and DI would significantly enhance the diagnostic capabilities of laboratories in detecting clinically relevant aneuploidies, thereby facilitating improved risk stratification and the selection of more appropriate treatment protocols.

## Supporting information

S1 File Raw dataset used in this analysis. (XLSX)

S1 Table Immunophenotypic characteristics by sex. (PDF)

S2 Table Association between HHD and the presence of cytogenetic abnormalities. (PDF)

S3 Table Cytogenetic and molecular rearrangements detected. (PDF)

S4 Table Diagnostic performance of DNA index across age groups. (PDF)

S5 Table Association between culture failure and DNA index. (PDF)
