# Supplementary material for: Diagnostic performance of DNA index for detection of high hyperdiploidy in childhood B-cell acute lymphoblastic leukemia
Source: PLoS One. 2026 Apr 20;21(4):e0347201. doi: 10.1371/journal.pone.0347201 (PMC13094976; doi:10.1371/journal.pone.0347201)
Supplement: S1 Table — (PDF) [file pone.0347201.s002.pdf]

S1 Table. Immunophenotypic characteristics by sex.

	Sex		
	**Female**	**Male**	**p**
	**n (%)**	**n (%)**	
**Age (groups)**			0.717
< 1 year	4 (44.4)	5 (55.6)	
1 to 10 years	65 (41.9)	90 (58.1)	
> 10 years	16 (34.8)	30 (65.2)	
**Maturation stage**			0.738
Common-B	66 (41.8)	92 (58.2)	
Pre-B	14 (35.0)	26 (65.0)	
Pro-B	5 (41.7)	7 (58.3)	
**CD123 expression**			0.176
Negative	23 (33.3)	46 (66.7)	
Positive	61 (44.2)	77 (55.8)	
**CD66c expression**			0.386
Negative	29 (36.3)	51 (63.8)	
Positive	56 (43.1)	74 (56.9)	
**CD123/CD66c co-expression**			0.157
Negative	36 (35.3)	66 (64.7)	
Positive	48 (45.7)	57 (54.3)	
**Blast size***	1.18 (1.06-1.37)	1.17 (1.06-1.30)	0.339
**DNA index***	1.03 (1.01-1.15)	1.02 (1.00-1.07)	0.355
**Chromosomes***	46 (46 - 47)	46 (46 - 47)	0.597

* Median (IQR), IQR: interquartile range (p25-p75).
