# Supplementary material for: Diagnostic performance of DNA index for detection of high hyperdiploidy in childhood B-cell acute lymphoblastic leukemia
Source: PLoS One. 2026 Apr 20;21(4):e0347201. doi: 10.1371/journal.pone.0347201 (PMC13094976; doi:10.1371/journal.pone.0347201)
Supplement: S2 Table — (PDF) [file pone.0347201.s003.pdf]

S2 Table. Association between HHD and the presence of cytogenetic abnormalities.

	Karyotype		
	≤50 chromosomes	>50 chromosomes	p
	n (%)	n (%)	
**Presence of structural abnormalities**			0.007
No	76 (89.4)	9 (10.6)	
Yes	52 (72.2)	20 (27.8)	
**Presence of molecular abnormalities**			0.003
No	91 (76.5)	28 (23.5)	
Yes	35 (97.2)	1 (2.8)	
