# Supplementary material for: Diagnostic performance of DNA index for detection of high hyperdiploidy in childhood B-cell acute lymphoblastic leukemia
Source: PLoS One. 2026 Apr 20;21(4):e0347201. doi: 10.1371/journal.pone.0347201 (PMC13094976; doi:10.1371/journal.pone.0347201)
Supplement: S3 Table — (PDF) [file pone.0347201.s004.pdf]

S3 Table. Cytogenetic and molecular rearrangements detected.

	n (%)
Hyperdiploidy (>50 chromosomes)	29 (18.5)
Hypodiploidy (<44 chromosomes)	1 (0.6)
t(9;22) / BCR-ABL	7 (4.5)
t(11q23) / KMT2A	3 (1.9)
t(1;19) / TCF3-PBX1	12 (7.6)
t(5;14) / IGH/IL3	1 (0.6)
t(12;21) / ETV6-RUNX1	16 (10.2)
No recurrent rearrangements detected	88 (56.1)
