# Supplementary material for: Diagnostic performance of DNA index for detection of high hyperdiploidy in childhood B-cell acute lymphoblastic leukemia
Source: PLoS One. 2026 Apr 20;21(4):e0347201. doi: 10.1371/journal.pone.0347201 (PMC13094976; doi:10.1371/journal.pone.0347201)
Supplement: S4 Table — (PDF) [file pone.0347201.s005.pdf]

**S4 Table. Diagnostic performance of DNA index across age groups.**

**<5 Years (n=65).**

| | DNA index | |
|---|---|---|
| **Karyotype** | **<1.10** | **≥1.10** |
| ≤50 chromosomes | 43 | 7 |
| >50 chromosomes | 1 | 14 |

| **Parameter** | **Value** | **(95% CI)** |
|---|---|---|
| Sensitivity (%) | 93.3 | (87.3-99.4) |
| Specificity (%) | 86.0 | (77.6-94.4) |
| PPV (%) | 66.7 | (55.2-78.1) |
| NPV (%) | 97.7 | (94.1-100.0) |
| LR+ | 6.67 | (3.31-13.43) |
| LR- | 0.08 | (0.01-0.52) |
| AUC | 0.93 | (0.86-0.99) |
| Accuracy | 0.88 | (0.77-0.95) |
| Kappa statistic | 0.70 | (0.51-0.89) |
| Youden index | 0.79 | - |

**5-10 Years (n=57).**

| | DNA index | |
|---|---|---|
| **Karyotype** | **<1.10** | **≥1.10** |
| ≤50 chromosomes | 39 | 6 |
| >50 chromosomes | 0 | 12 |

| **Parameter** | **Value** | **(95% CI)** |
|---|---|---|
| Sensitivity (%) | 100.0 | (73.5-100.0) |
| Specificity (%) | 86.7 | (77.8-95.5) |
| PPV (%) | 66.7 | (54.4-78.9) |
| NPV (%) | 100.0 | (91.0-100.0) |
| LR+ | 7.50 | (3.56-15.80) |
| LR- | 0.00 | - |
| AUC | 0.93 | (0.86-0.99) |
| Accuracy | 0.88 | (0.77-0.95) |
| Kappa statistic | 0.73 | (0.54-0.93) |
| Youden index | 0.87 | - |

**>10 Years (n=35).**

| | DNA index | |
|---|---|---|
| **Karyotype** | **<1.10** | **≥1.10** |
| ≤50 chromosomes | 33 | 0 |
| >50 chromosomes | 0 | 2 |

| **Parameter** | **Value** | **(95% CI)** |
|---|---|---|
| Sensitivity (%) | 100.0 | (15.8-100.0) |
| Specificity (%) | 100.0 | (89.4-100.0) |
| PPV (%) | 100.0 | (15.8-100.0) |
| NPV (%) | 100.0 | (89.4-100.0) |
| LR+ | - | - |
| LR- | 0.00 | - |
| AUC | 1.00 | (0.72-1.00) |
| Accuracy | 1.00 | (0.90-1.00) |
| Kappa statistic | 1.00 | (0.63-1.00) |
| Youden index | 1.00 | - |

VPP: positive predictive value, VPN: negative predictive value, LR+: positive likelihood ratio, LR-: negative likelihood ratio, AUC: area under the curve, 95% CI: 95% confidence interval.
