# Supplementary material for: Diagnostic performance of DNA index for detection of high hyperdiploidy in childhood B-cell acute lymphoblastic leukemia
Source: PLoS One. 2026 Apr 20;21(4):e0347201. doi: 10.1371/journal.pone.0347201 (PMC13094976; doi:10.1371/journal.pone.0347201)
Supplement: S5 Table — (PDF) [file pone.0347201.s006.pdf]

S5 Table. Association between culture failure and DNA index.

	Culture		
	**Success**	**Failure**	**p**
	**n (%)**	**n (%)**	
**DNA index**			0.858
<1.10	116 (73.9)	38 (71.7)	
≥1.10	41 (26.1)	15 (28.3)	
**DNA index***	1.07 (1.00-1.13)	1.07 (1.00-1.15)	0.671

* Median (IQR), IQR: interquartile range (p25-p75).
